# THE SPLENIC INDEX AS PREDICTOR OF BLEEDING AND VARICEAL RECURRENCE IN THE LATE FOLLOW-UP OF SCHISTOSOMOTIC PATIENTS AFTER EXCLUSIVE ENDOSCOPIC TREATMENT

**DOI:** 10.1590/0102-672020210002e1638

**Published:** 2022-01-31

**Authors:** Alexandre BORGHERESI, Ramiro COLLEONI, Milton SCALABRINI, David SHIGUEOKA

**Affiliations:** 1 Universidade Federal de São Paulo - UNIFESP, Cirurgia Digestiva, São Paulo, SP, Brasil; 2 Universidade Federal de São Paulo - UNIFESP, Radiologia, São Paulo, SP, Brasil

**Keywords:** Schistosomiasis, Hypertension, Portal, Esophageal and Gastric Varices, Hypersplenism, Endoscopy, Gastrointestinal, Esquistossomose, Hipertensão Portal, Varizes Esofágicas e Gástricas, Hiperesplenismo, Endoscopia Gastrointestinal

## Abstract

Endoscopic treatment for esophageal variceal has been used as the main intervention in patients with portal hypertension secondary to schistosomiasis, but with significant rates of recurrence of esophageal variceal and rebleeding. The long-term results of exclusive endoscopic treatment are poorly studied as the relationship of the splenic dimensions in this context.

**AIM:**

The aim of this study was to identify, through ultrasonography, whether the splenic index and the longitudinal (craniocaudal) dimension of the spleen are the predictors of rebleeding and variceal recurrence in late follow-up of patients with nonoperated schistosomiasis, after endoscopic eradication of esophageal variceal.

**METHODS:**

This is a retrospective and observational study analyzing the medical records of patients diagnosed with hepatosplenic schistosomiasis. The receiver operating characteristic curve was used to determine the best cutoff point for the mean splenic index as a predictor of recurrence and bleeding.

**Results:**

A follow-up of 54 patients were analyzed during the period from 2002 to 2018. The mean follow-up time was 8 years. The splenic index with value >144 was proved to be a sensitive test for rebleeding. In the analysis of the longitudinal dimension, the spleen length of >20 cm showed a statistically significant test for recurrence of variceal and a length >19 cm presented as a very sensitive and statistically significant test for rebleeding.

**CONCLUSION:**

Splenic index and craniocaudal dimension analysis, obtained by ultrasonography, can predict recurrence of varicose veins and rebleeding after exclusive endoscopic treatment.

## RESUMO - RACIONAL

O tratamento endoscópico das varizes esofágicas tem sido utilizado como principal intervenção em pacientes com hipertensão portal secundária à esquistossomose, mas com taxas significativas de recorrência de varizes esofágicas e ressangramento. Os resultados em longo prazo do tratamento endoscópico exclusivo são pouco estudados quanto à relação das dimensões esplênicas neste contexto.

**OBJETIVO:**

Avaliar, por meio da ultrassonografia, o *índice esplênico* e a dimensão longitudinal (craniocaudal) do baço como preditores de ressangramento e recorrência de varizes no seguimento tardio de pacientes esquistossomóticos não operados, após erradicação endoscópica das varizes esofágicas.

**MÉTODOS:**

Estudo observacional retrospectivo por meio da análise de prontuários de pacientes com diagnóstico de esquistossomose hepatoesplênica. A curva ROC foi usada para determinar o melhor ponto de corte para o índice esplênico médio como preditor de recorrência e sangramento.

**RESULTADOS:**

Foram analisados 54 pacientes, durante o período de 2002 a 2018. O tempo médio de seguimento foi de 8 anos. O *índice esplênico* provou ser um teste sensível em valores acima de 144 como preditor de ressangramento. Na análise da dimensão longitudinal, o valor acima de 20 cm apresentou teste estatisticamente significativo para recorrência de varizes e valor acima de 19 cm apresentou-se como teste muito sensível e estatisticamente significativo para ressangramento.

**CONCLUSÃO:**

A análise do *índice esplênico* e da dimensão craniocaudal, obtidos por ultrassonografia, podem predizer recorrência de varizes e ressangramento após erradicação endoscópica exclusiva.

## INTRODUCTION

Schistosomiasis is an acute and chronic parasitic disease caused by trematode worms of the genus *Schistosoma*. Estimates show that annually 220.8 million people in the world need preventive treatment^10^ ^,^ ^14^ ^,^ ^24^. In Brazil, parasitosis is exclusively caused by *Schistosoma mansoni* and it represents an important impact on public health, accounting for 4 million people infected and 20 million people exposed to infection^14^. The most severe form is hepatosplenic schistosomiasis, which is considered an important cause of morbidity and mortality in 3-10% of those infected^8^ ^,^ ^11^ ^,^ ^21^. Chronic hepatosplenic involvement describes a wide range of important clinical manifestations, with portal hypertension being the main cause of morbidity and mortality in these patients. With the chronic evolution of the disease, these patients may develop ascites, splenomegaly, portal thrombosis, and esophageal and stomach variceal, with upper digestive hemorrhage being the major cause of mortality^2^ ^,^ ^3^ ^,^ ^4^ ^,^ ^15^ ^,^ ^18^. For this reason, the development of methods for early diagnosis of complications and effective treatment is the priority in the current medical literature.

Endoscopic treatment of esophageal variceal has been used as the main intervention in patients with portal hypertension secondary to schistosomiasis. The evaluation of the long-term results of exclusive endoscopic treatment in this group of patients is still insufficient and controversial. Studies have already demonstrated the importance of abdominal ultrasound findings with Doppler in the diagnosis of schistosomiasis in addition to the adequate reproducibility of this method; however, there is still a lack of information on the prognostic value and clinical applicability of these findings. Thus, it is important to study the etiology of this disease and the values of the splenic index as a predictor of response to treatment in order to improve the quality of care and reduce mortality and morbidity.

The aim of this study was to evaluate the long-term follow-up of patients with hepatosplenic schistosomiasis who underwent exclusive endoscopic treatment with the eradication of esophageal variceal in terms of the ultrasound dimensions of the spleen and their relationship with esophageal variceal recurrence and rebleeding.

## METHODS

### Study sample and design

This is an observational, retrospective study, analyzing the medical records of patients diagnosed with hepatosplenic schistosomiasis who were followed up at the Outpatient Clinic of the Surgical Gastroenterology Group at Escola Paulista de Medicina - UNIFESP, from January 1, 2002 to January 1, 2019.

The patients who underwent exclusive endoscopic treatment with eradication of esophageal variceal by elastic ligation and/or sclerotherapy and who were followed up once yearly after the eradication of variceal with ultrasound, upper gastrointestinal endoscopy, laboratory tests, and clinical history were included in this study. This study was conducted in accordance with the Declaration of Helsinki and was approved by the Research Ethics Committee of UNIFESP (n = 2,462,087).

The eligibility criteria included patients with minimum 12 months follow-up after eradication of esophageal variceal detected by endoscopic examination and the availability of laboratory tests, endoscopic ultrasound, and Doppler abdominal series. Patients with liver disease of another etiology, those who underwent surgery (splenectomy) before endoscopic eradication, and those not having longitudinal dimension report or the splenic index in ultrasound examinations were excluded from this study.

### Variables

Epidemiological data were collected in a standardized way and then tabulated. All patients were retrospectively evaluated for Doppler ultrasonography, upper digestive endoscopy, laboratory tests, and clinical history, which were performed serially during the follow-up period.

The ultrasound information was evaluated and tabulated according to the Niamey protocol, adopted as a standardization by the World Health Organization (WHO) in the evaluation of examinations of patients with schistosomotis^1^ ^,^ ^3^. The parameters such as gauge and flow of the portal vein and splenic vein, cavernomatous transformation, splenic index, the presence of siderotic nodules (Gamna-Gandy bodies), and size of the spleen and ascites were evaluated. In relation to laboratory tests, the following factors were evaluated: leukogram, hemoglobin, bilirubin, international normalized ratio (INR), activated partial thromboplastin time (aPTT), albumin, platelet count, and liver enzymes. Hemoglobin values <12 g/l, leukocytes <3,500, glutamic oxaloacetic transaminase (TGO) >32, glutamic pyruvic transaminase (TGP) >33, albumin <3.5, international normalized ratio (INR) >1.2, platelets <150,000, and total bilirubin >1 were considered altered^19^.

The endoscopic data such as recurrence of esophageal variceal, caliber and number of varicose veins in recurrence, need for new treatment sessions after eradication, type of treatment performed, and rebleeding were analyzed. The clinical evolution parameters such as mortality and need for surgery for portal hypertension during follow-up and bleeding after eradication were evaluated.

### Statistical analysis

For the descriptive analysis, frequency and percentage were used as categorical variables and mean, standard deviation (SD), minimum, median, and maximum were used as continuous variables. For the analysis of correlation and receiver operating characteristic (ROC) curve, the mean value of the splenic index and longitudinal dimension of each patient was used. Patients who did not have an ultrasound assessment of the splenic index value or the longitudinal dimension of the spleen were excluded from these analyses. To determine the best cutoff point for the mean splenic index that predicted recurrence and bleeding, the ROC curve was used. Accuracy was determined from the area under the ROC curve (AUC) using the SPSS version 17.0 program. Sensitivity, specificity, positive predictive value (PV+), and negative predictive value (PV−) of the variables indicative of recurrence and bleeding were analyzed. To compare the splenic index with categorical variables, the t test was used. The statistical analysis was performed using the SAS version 9.1 program, with a significance level of 5%.

## RESULTS

The medical records of 44 patients (female = 22; male = 22) were analyzed, including a description of the splenic index and/or the longitudinal dimension of the spleen. The mean age of patients at time zero was 49.75 years, a minimum of 31 and a maximum of 79 years. The average follow-up time was 8.07 years, with a minimum of 1 and a maximum of 16 years (Table 1). Regarding the examinations, a total of 150 abdominal Doppler ultrasounds, 245 upper digestive endoscopies, and 76 laboratory tests were analyzed.

**Table 1 -:** Distribution of age and follow-up time of patients with schistosomiasis with esophageal variceal eradicated by exclusive endoscopic treatment.

	Total
Age at eradication (years)	
Average (SD)	49.75 (11.61)
Median	48
Minimum-maximum	31-79
Total	44
Follow-up time (years)	
Average (SD)	8.07 (12.51)
Median	6
Minimum-maximum	1-16
Total	44

Regarding the primary outcomes, 63.6% presented recurrence of esophageal variceal after eradication. The average time for the first relapse was 2.32 years, with an average of 2.07 relapses in the follow-up period (Table 2). Notably, 29.5% of the patients had gastrointestinal bleeding after eradication and 76.9% of the cases had bleeding with hemodynamic or hematimetric repercussions.

**Table 2 -:** Description of endoscopic findings in relation to variceal relapse in patients with schistosomiasis with esophageal variceal eradicated by exclusive endoscopic treatment.

	Total
Relapse, n (%)	
No	16 (36.4)
Yes	28 (63.6)
Total	44
Recurrence quantity	
Average (SD)	2.07 (1.54)
Median	1
Minimum-maximum	1-6
Total	28
Average number of variceal in relapse	
Average (SD)	2.42 (1.01)
Median	2.325
Minimum-maximum	1-6
Total	28
Time to first relapse (years)	
Average (SD)	2.32 (2.36)
Median	1
Minimum-maximum	1-9
Total	28

The most commonly used therapy for both eradication control after recurrence and bleeding was elastic ligation (Table 3).

**Table 3 -:** Description of endoscopic findings in relation to rebleeding in patients with schistosomiasis with esophageal variceal eradicated by exclusive endoscopic treatment.

	Total
Bleeding, n (%)	
No	31 (70.5)
Yes	13 (29.5)
Total	44
Type of treatment, n (%)	
Elastic bandage	21 (8.6)
Sclerotherapy	13 (5.3)
Total	245
Repercussion, n (%)	
No	3 (23)
Yes	10 (76.9)

The mortality was 6.8% and the patients died secondary to digestive bleeding during follow-up. The other causes are shown in Table 4.

**Table 4 -:** Description of mortality during follow-up of patients with schistosomiasis with esophageal variceal eradicated by exclusive endoscopic treatment.

	Total
Mortality, n (%)	
No	34 (77.3)
Yes	10 (22.7)
Total	44
Causes of death, n (%)	
Postoperative bleeding-abdominal	1
Unknown	3
Digestive bleeding	3 (30)
Congestive heart failure	1
Nosocomial pneumonia	1
Spontaneous bacterial peritonitis	1
Total	10
Digestive bleeding mortality, n (%)	
No	41 (93.2)
Yes	3 (6.8)
Total	44

The descriptive analysis of the ultrasound findings showed that the average caliber of the portal vein was 1.39 cm and that of the splenic vein was 1.26 cm. The presence of ascites was identified in 14.7% of the examinations, 59% of which were small (Table 5).

**Table 5 -:** Description of ultrasound findings in patients with schistosomiasis with esophageal variceal eradicated by exclusive endoscopic treatment.

	Total
Portal gauge	
Average (SD)	1.39 (0.38)
Median	1.4
Minimum-maximum	0.4-2.4
Total	44
Caliber of splenic vein	
Average (SD)	1.26 (0.38)
Median	1.2
Minimum-maximum	0.6-2.4
Total	44
Ascites, n (%)	
No	128 (85.3)
Yes	22 (14.7)
Total	150
Ascites-volume, n (%)	
Great	2 (9)
Moderate	7 (31.8)
Little	13 (59)
Total	22

Hepatofugal flow was observed in 9% of patients. The size of the spleen (longitudinal) was described in 23 patients, with a mean value of 20.16 cm. The splenic index was described in 40 patients, with a mean value of 151.6. Cavernomatous transformation was identified in 38.6% of patients. Notably, 47.7% of the patients had siderotic nodules on ultrasound findings (Table 6).

**Table 6 -:** Description of ultrasound findings in schistosomal patients with esophageal variceal eradicated by exclusive endoscopic treatment.

	Total
Hepatofugal, n (%)	
No	39 (88.6)
Yes	4 (9)
Total	44
Average size of the spleen-longitudinal	
Average (SD)	20.16 (2.63)
Median	20
Minimum-maximum	15-24.2
Total	23
Average splenic index	
Average (SD)	151.62 (41.91)
Median	142.25
Minimum-maximum	76-221.5
Total	40
Cavernomatous transformation, n (%)	
No	27 (61.3)
Yes	17 (38.6)
Total	44
Siderotic nodule, n (%)	
No	23 (52.2)
Yes	21 (47.7)
Total	44

The laboratory findings showed the mean value of the leukogram was 4,152, with 57.1% of patients with leukopenia. The mean platelet value was 73,333, with 88% of patients with thrombocytopenia. Also, 28.6% of the patients had anemia (Table 7).

**Table 7 -:** Description of laboratory findings in schistosomal patients with esophageal variceal eradicated by exclusive endoscopic treatment.

	Total
Leukogram	
Average (SD)	4152.38 (2395.98)
Median	3,235
Minimum-maximum	1,490-11,600
Total	44
Interpretation, n (%)	
Normal	18 (42.9)
Not normal	24 (57.1)
Platelets	
Average (SD)	73333.33 (58981.46)
Median	53,000
Minimum-maximum	18,000-241,000
Total	44
Interpretation, n (%)	
Normal	5 (11.9)
Not normal	37 (88.1)
Bilirubin	
Average (SD)	1.6 (1.26)
Median	1.16
Minimum-maximum	0.4-4.8
Total	35
Interpretation, n (%)	
Normal	14 (40)
Not normal	21 (60)
INR	
Average (SD)	1.37 (0.28)
Median	1.3
Minimum-maximum	1-2.22
Total	39
Interpretation, n (%)	
Normal	10 (25.6)
Not normal	29 (74.4)
Hemoglobin	
Average (SD)	12.04 (2.09)
Median	12
Minimum-maximum	15
Total	44
Interpretation, n (%)	
Normal	30 (71.4)
Not normal	12 (28.6)
TGO	
Average (SD)	39.63 (23.46)
Median	33
Minimum-maximum	16-150
Total	38
Interpretation, n (%)	
Normal	19 (50)
Not normal	19 (50)
TGP	
Average (SD)	32.78 (10.65)
Median	32
Minimum-maximum	60
Total	37
Interpretation, n (%)	
Normal	22 (59.5)
Not normal	15 (40.5)
Albumin	
Average (SD)	3.55 (0.87)
Median	3.7
Minimum-maximum	0.7-4.9
Total	35
Interpretation, n (%)	
Normal	22 (62.9)
Not normal	13 (37.1)

**Figure 1. -:**
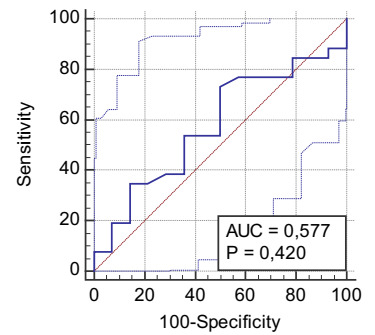
ROC curve showing mean splenic index for variceal recurrence in schistosomal patients with esophageal variceal eradicated by exclusive endoscopic treatment.

The ROC curve of the average splenic index for recurrence of varicose veins had a cutoff value of >169, with a VP+ of 73% (AUC = 0.57, p = 0.42) (Figure 1 and Table 8).

**Table 8 -:** Diagnostic test of mean splenic index for variceal relapse in patients with schistosomiasis with esophageal variceal eradicated by exclusive endoscopic treatment.

	Relapse, n (%)		Total
	Yes	No	
Splenic index, n (%)			
Yes (>169)	19 (47.5)	7 (17.5)	26 (65)
No (≤169)	7 (17.5)	7 (17.5)	14 (35)
Total	26 (65)	14 (35)	40 (100)

**Table:** 

VP+	0.73
VP−	0.50
Sensitivity	0.73
Specificity	0.50
Accuracy	0.65
Prevalence	0.65

**Figure 2 -:**
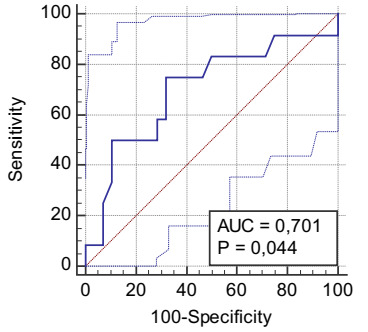
ROC curve showing mean splenic index for bleeding in schistosomal patients with esophageal varices eradicated by exclusive endoscopic treatment.

The ROC curve of the mean splenic index for bleeding had a cutoff point of >144, with a sensitivity of 75% and VP− of 86% (AUC = 0.70, p = 0.044) (Figure 2 and Table 9).

**Table 9 -:** Diagnostic test of mean splenic index for bleeding in patients with schistosomiasis with esophageal variceal eradicated by exclusive endoscopic treatment.

	Blood loss, n (%)		Total
	Yes	No	
Splenic index, n (%)			
Yes (>140)	9 (22.5)	9 (22.5)	18 (45)
No (≤140)	3 (7.5)	19 (47.5)	22 (55)
Total	12 (30)	28 (70)	40 (100)

**Table:** 

VP+	0.50
VP−	0.86
Sensitivity	0.75
Specificity	0.68
Accuracy	0.70
Prevalence	0.30

**Figure 3 -:**
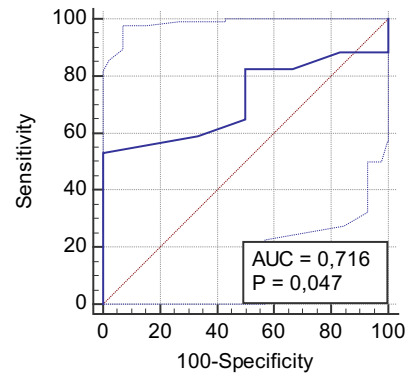
ROC curve showing mean spleen size for relapse in patients with schistosomiasis with esophageal variceal eradicated by exclusive endoscopic treatment.

The ROC curve of the mean size of the spleen due to variceal recurrence gave a cutoff value of >20, with a specificity of 100% and VP+ of 100% (AUC = 0.71, p = 0.047) (Figure 3 and Table 10).

**Table 10 -:** Diagnostic test of mean spleen size for varicose relapse in patients with schistosomiasis with esophageal variceal eradicated by exclusive endoscopic treatment.

	Relapse, (%)		Total
	Yes	No	
Size, n (%)			
Yes (>20)	9 (39.1)	0 (0)	9 (39.1)
No (≤20)	8 (34.8)	6 (26.1%)	14 (60.9)
Total	17 (73.9)	6 (26.1)	23 (100)

**Table:** 

VP+	1.00
VP−	0.43
Sensitivity	0.53
Specificity	1.00
Accuracy	0.65
Prevalence	0.74

**Figure 4 -:**
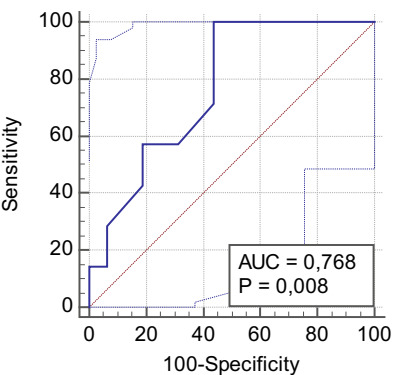
ROC curve showing mean spleen size for bleeding in schistosomal patients with esophageal variceal eradicated by exclusive endoscopic treatment.

The average spleen ROC curve for bleeding had a cutoff value of >19, with a sensitivity of 100% and VP− of 100% (AUC = 0.76, p = 0.008) (Figure 4 and Table 11).

**Table 11 -:** Diagnostic test of mean spleen size for bleeding in schistosomal patients with esophageal variceal eradicated by exclusive endoscopic treatment.

	Blood loss, n (%)		Total
	Yes	No	
Size, n (%)			
Yes (>19)	7 (30.4)	7 (30.4)	14 (60.9)
No (≤19)	0 (0)	9 (39.1)	9 (39.1)
Total	7 (30.4)	16 (69.6)	23 (100)

**Table:** 

VP+	0.50
VP−	1.00
Sensitivity	1.00
Specificity	0.56
Accuracy	0.70
Prevalence	0.30

**Figure 5 -:**
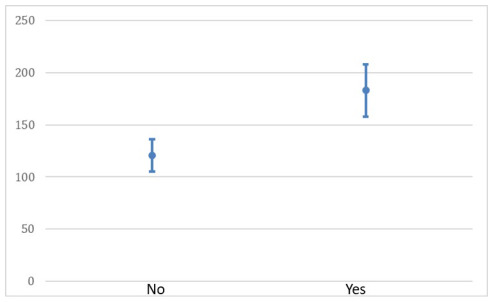
Relationship of splenic index with indication for surgery in schistosomal patients with esophageal variceal eradicated by exclusive endoscopic treatment.

Finally, the correlation of categorical variables and splenic index showed a significant difference in the splenic index by surgery. Patients who underwent surgery had a higher mean index (p = 0.0021). There was no statistically significant difference in the other variables studied (Table 12, Figure 5).

**Table 12 -:** Correlation of categorical variables with the mean splenic index of schistosomal patients with esophageal variceal eradicated by exclusive endoscopic treatment.

	Average splenic index				p-value
	N	Average (SD)	Median	Minimum-Maximum	
Relapse					
No	16	159.06 (38.81)	161.5	86-210	0.4170
Yes	28	147.61 (43.69)	133.5	76-221.5	
Total	44	151.62 (41.91)	142.25	76-221.5	
Surgery					
No	38	120.74 (19.47)	126	94.7-144	0.0021
Yes	6	182.92 (31.54)	189.75	130-220	
Total	44	151.83 (40.97)	138.5	94.7-220	
Bleeding					
No	31	143.53 (39.05)	132.75	80-220.75	0.0614
Yes	13	170.48 (43.94)	183.5	76-221.5	
Total	44	151.62 (41.91)	142.25	76-221.5	
Repercussion					
No	3	133.4 (44.5)	130	76-199.5	0.1837
Yes	10	163.66 (40.3)	163	101.3-221.5	
Total	13	155.26 (42.51)	145.75	76-221.5	
Digestive bleeding mortality					
No	41	148.48 (41.65)	134	76-221.5	0.0966
Yes	3	190.33 (24.42)	198	163-210	
Total	44	151.62 (41.91)	142.25	76-221.5	

## DISCUSSION

The main finding of this study is that the splenic index and one-dimensional craniocaudal analysis of the spleen, obtained by ultrasonography, can predict the recurrence of esophageal variceal and rebleeding after exclusive endoscopic eradication, contributing to the therapeutic planning of schistosomal patients with portal hypertension.

Schistosomiasis is endemic over a vast area of the country, and it is considered a serious and neglected public health problem because it affects millions of people, thereby causing an expressive number of serious forms and deaths^13^. Reducing the morbidity and mortality of schistosomiasis requires early detection and prompt treatment of all carriers to prevent the accumulative pathogenic action of *S. mansoni* eggs from causing changes in affected organs. However, only few places have a reference center for the treatment and follow-up of these patients.

This study was carried out at the Outpatient Clinic of University Hospital and the longitudinal follow-up of this disease was performed by specialists.

The limited number of patients is due to the strict inclusion criteria of this study. Only patients with eradicated varicose veins who had not undergone splenectomy at the beginning of the proposed follow-up were evaluated. However, they were evaluated retrospectively for a long period of follow-up, with an average duration of 8 years, making it possible to collect periodic information from a significant number of laboratories, endoscopic, and ultrasound examinations.

As demonstrated in literature, there is no difference between genders, also indicated by this study with an equal proportion between genders^13^ ^,^ ^16^. The compensated hepatosplenic form predominates in adolescents and young adults aged between 10 and 30 years. This study showed a higher average age, which is due to the fact that a group of patients who had been in clinical follow-up for some time, with varicose veins eradicated and compensated in the initial analysis time, was selected. Age at diagnosis was not taken into account.

Upper gastrointestinal bleeding secondary to variceal rupture is one of the main complications of portal hypertension due to schistosomiasis, occurring in approximately 30-40% of patients^13^ ^,^ ^20^. Despite advances in endoscopic therapy, the mortality rate for a single episode of variceal bleeding is 20%^5^ ^,^ ^13^. In this study, 6.8% of the patients followed up died of digestive hemorrhage after eradicating the variceal. For the secondary prevention of variceal hemorrhage in patients with schistosomal portal hypertension, we have endoscopy as the most performed technique, with reported efficacy of sclerotherapy or endoscopic elastic ligation ranging from 54% to 82.3%^7^. In our sample, elastic ligation was the most used technique for both eradication and bleeding control.

Even with endoscopic advances, according to the scarce data in the literature on the subject, we found a rate of up to 62% in recurrence of esophageal variceal after endoscopic treatment and a bleeding rate of 46%^9^ ^,^ ^22^. In our series, the data are similar in relation to recurrence (63.6%) and lower in terms of rebleeding (29.5%). However, it is important to highlight that most patients with rebleeding had hematimetric or hemodynamic repercussions. We did not find in the literature, for a comparative analysis, studies that specifically assess the characteristics of recurrence, as demonstrated in our research. However, we considered 2.32 years of mean variceal-free interval to be a short time and an expressive 2.07 recurrence episodes on average per follow-up period.

According to the current technical guidelines of the Ministry of Health, surgical treatment can be considered in the following situations: (1) varicose veins with signs of impending bleeding at endoscopy; (2) large gastric and esophageal variceal in patients who live outside large medical centers; (3) persistent large gastric variceal after endoscopic eradication of esophageal variceal; and (4) hypersplenism with disabling clinical manifestation^14^. In other conditions, patients should be evaluated periodically, and in cases of unfavorable evolution, complementary surgical treatment would be indicated.

During the follow-up evaluated in our study, 13% of patients underwent splenectomy following the criteria described above. These patients who underwent surgery had higher values of splenic index (p = 0.0021), demonstrating a positive correlation between the dimensions of the spleen and the need for the indication of the procedure (Table 12 and Figure 5). The finding of a significant increase in splenic volume could be considered a criterion to justify surgical treatment in these patients using an azigoportal disconnection procedure or distal splenorenal shunt. We did not find in the literature studies that analyze the rate of surgical indication in patients with varicose veins eradicated for a comparative analysis.

The changes observed in the results of laboratory tests to assess liver function have not been explored because they are not related to the objective in the research, however, we can analyze that a considerable part of the patients presented changes in these tests, inferring a late evolution to the decompensated hepatosplenic form.

Using the dimensions of the spleen, we sought to identify predictors of variceal recurrence and bleeding, the main causes of morbidity and mortality in this environment. The splenic index with value >144 was proved to be a sensitive test for bleeding (PV− 85%, AUC = 0.70, p = 0.044) and can be a useful test when splenic index is <144 (negative test). Regarding variceal recurrence, the splenic index >169 proved to be a nonspecific test, with a PV+ of 73%, without statistical significance. However, in the analysis of the craniocaudal dimension, the spleen length of >20 cm is proved to be a very specific and statistically significant test for recurrence (VP+ 100%, AUC = 0.71, p = 0.047). As a result, the value >20 cm (positive test) is a good predictor of recurrence. Similarly, the spleen length of >19 cm is a very sensitive and significantly statistical test for bleeding (VP−100%, AUC = 0.76, p = 0.008). The spleen length of <19 cm (negative test) is an accurate test to rule out bleeding.

Due to the lack of evidence regarding exclusive long-term endoscopic therapy, some more current research seeks to assess the benefits of associated splenectomy. Case series indicate that the combination of surgical and endoscopic therapy may be more effective than using exclusive therapy^6^ ^,^ ^17^. Following this hypothesis, a retrospective study showed that endoscopic sclerotherapy to control relapse and eradication was more effective in patients who had already undergone surgical treatment^12^ ^,^ ^17^ ^,^ ^23^. Costa Lacet et al.^4^ in a prospective and randomized study demonstrated greater success in eradication and relapse control in patients undergoing combined surgical and endoscopic therapy. However, studies were conducted with the aim of evaluating efficacy of eradication and control of bleeding recurrence at an early stage of treatment. We do not have similar studies evaluating late outcomes in the population with varicose veins previously eradicated.

This study also has limitations. It is a retrospective and observational study with a limited population. A specific group of patients who had the characteristic of adhering to the treatment and the long follow-up were selected. In general, patients with schistosomiasis have social characteristics that hinder this adherence, originate from endemic areas outside urban centers, have low income and low education, and often change their address and professional activity. For these reasons, we may have presented selection bias. There was also gauging bias due to the absence of the cutoff value of the splenic index or craniocaudal dimension in some evaluated exams. To minimize information bias, all data were collected in a standardized way and arranged in tabular format by only one evaluator.

Schistosomiasis is a disease that is still prevalent and relevant to public health in our country. The current literature is scarce in relation to the late follow-up of these patients, and less is known about the long-term consequences of exclusive endoscopic treatment. The studies are still controversial in relation to the maintenance of the spleen, its progressive growth, and the clinical repercussions resulting from it. Periodic analysis of ultrasound examinations is recommended in the literature and practiced in our country; however, its clinical applicability is lacking. Therefore, prospective and randomized studies should be carried out to define the best follow-up for these patients.

## CONCLUSIONS

The splenic index and one-dimensional craniocaudal analysis of the spleen, obtained by ultrasound, can predict the recurrence of esophageal variceal and rebleeding after exclusive endoscopic eradication, contributing to the therapeutic planning of schistosomal patients with portal hypertension.
